# Dual Effect of Solution pH on Ammonia Recovery in Membrane Distillation – Influence on pH Partitioning and Mass Transfer Coefficient

**DOI:** 10.1021/acsestengg.5c00332

**Published:** 2025-07-28

**Authors:** Kai Yang, Benjamin Michael Simplot, Mohan Qin

**Affiliations:** Department of Civil and Environmental Engineering, 5228 University of Wisconsin−Madison, Madison, Wisconsin 53706, United States

**Keywords:** ammonia recovery, membrane distillation, pH, ammonia transport, mass transfer coefficient

## Abstract

Recovering ammonia from wastewater by membrane distillation (MD) is a sustainable approach to remediating environmental issues while simultaneously conserving energy both in wastewater treatment and in the Haber-Bosch process. MD leverages the volatility of ammonia to enhance ammonia transport, and hence its performance is impacted by the pH of the solution. We comprehensively investigated the effect of pH on ammonia transport and recovery efficiency using both experimental and simulation approaches. Our analyses provide new insights into how solution pH significantly impacts ammonia recovery through two primary mechanisms: it both governs the ammonia-ammonium equilibrium and influences the ammonia mass transfer coefficient. When changing MD feed solution pH from 9 to 10, ammonia flux is enhanced by 177% and ammonia mass transfer coefficient increases from 2.64 × 10^–6^ m·s^–1^ to 6.14 × 10^–6^ m·s^–1^. Notably, solution pH adjustment has a more significant effect than increasing solution temperature on enhancing the ammonia mass transfer coefficient and improving recovery efficiency, making it a more feasible and effective approach for improving ammonia transport and recovery. Additionally, our explicit simulations of ammonia recovery efficiency provide valuable insights for optimizing MD performance by adjusting solution pH values and operation time, and enable a maximum profit estimation of $598,000 for operating MD to recover ammonia in a dairy farm with 2000 cows.

## Graphical abstract

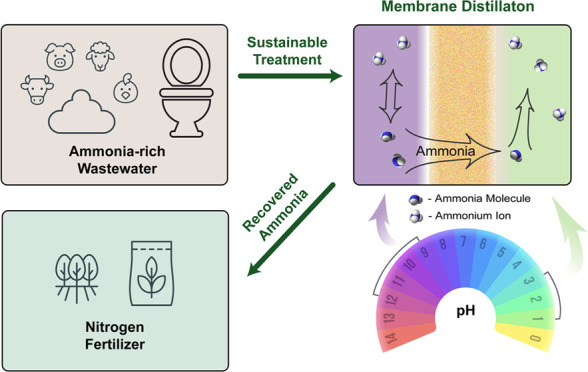

## 1 Introduction

As an essential chemical with massive demand in industry and agriculture, ammonia is predominantly produced by the energy-intensive Haber-Bosch process at the cost of up to 2% of global energy consumption and 5% of global carbon dioxide emission.^1,2^ Meanwhile, ammonia is also a major contaminant in ammonia-rich wastewater that potentially pollutes surface and groundwater, poses a threat to aquatic life, and aggravates climate change if wastewater is not adequately treated.^3–7^ Livestock wastewater, which has a total ammonia nitrogen (TAN) concentration of up to 7000 mg-N/L, causes nearly 60% of total ammonia emissions originating from natural and anthropogenic sources.^8,9^ Recovering ammonia from livestock wastewater offers a sustainable and efficient approach to addressing environmental concerns while reducing reliance on the Haber-Bosch process for ammonia production.

Originally developed for the desalination of hypersaline water, membrane distillation (MD) deploys a hydrophobic porous polymer membrane to transport volatile molecules across the membrane by evaporation and subsequent condensation.^10–14^ Because of the high volatility of ammonia, MD has been applied for the extraction of ammonia from hydrolyzed urine and livestock wastewater with a recovery rate of 195 g-N·m^–2^·h^–1^ and recovery efficiency up to 99%.^14–18^ MD is generally considered to have a lower fouling propensity than hydraulic pressure-driven membrane processes including reverse osmosis (RO) and nanofiltration (NF) due to its larger membrane pore size and low operating pressure, making it suitable for waste streams.^18,19^

Ammonia recovery in MD is highly influenced by the ammonia-ammonium equilibrium in aqueous solution, which is governed by both the dissociation constant p*K*_a_ and solution pH.^20^ The dissociation constant p*K*_a_ is temperature dependent and increasing temperature is an approach to lowering the p*K*_a_ value and converting ammonium ions to volatile ammonia, but the variation of p*K*_a_ is limited and heating up the solution demands massive energy. With a room temperature solution being heated to 60 °C, the p*K*_a_ decreases from 9.25 to 8.28.^11,21^ The limited variation in p*K*_a_ makes the concentration of volatile ammonia critically dependent on the solution pH value. In certain types of wastewater, ammonia molecules are a significant portion of TAN due to their high pH value.^22^ For instance, the pH of urine is around 9.0, with 36% of TAN existing as NH_3_ in the solution after hydrolysis.^23,24^ Extensive research has been conducted to enhance ammonia transport in the MD process, including increasing the solution temperature to increase ammonia volatility, boosting feed flow rate to enhance mixing conditions of boundary layers, and extending operation duration time.^16,17^ When it comes to the impact of pH, previous studies have primarily focused on the pH value in the feed solution. It has been demonstrated that increasing pH in the feed solution leads to a faster ammonia depletion rate and a higher ammonia mass transfer coefficient.^16^ Efforts to increase ammonia flux in MD have involved the use of an acidic collector solution to reduce the ammonia molecule concentration in the collector.^11^ Direct contact membrane distillation has been found to perform better in ammonia recovery from high pH aqueous solutions, and its excellent separation properties favor recovering ammonia from wastewater.^25^ However, these efforts have been implemented without extensive quantitative analysis to fully understand the impact of this approach on ammonia recovery efficiency. There exists a knowledge gap in how the solution pH in both the feed and collector sides impacts the ammonia mass transfer coefficient and overall MD performance. Therefore, a systematic evaluation of the influence of pH in both the feed and collector solutions on ammonia transport is essential to advance our understanding of MD and to optimize the MD process.

In this study, we evaluated the impact of pH in both the feed and collector solutions on ammonia transport in direct contact MD and developed a mathematical model to predict pH profiles and ammonia recovery efficiency. Specifically, we varied the initial pH values of the feed solution while maintaining a fixed initial pH in the collector solution, and vice versa, to thoroughly investigate the effects of pH on ammonia transport. The experimental results were utilized to calculate the ammonia mass transfer coefficients, providing insight into the critical role of pH in ammonia transport and enabling the prediction of ammonia recovery efficiency in MD based on solution pH and operation time. Then hydrolyzed cow urine was used to validate MD performance in ammonia recovery. We also conducted a preliminary profit analysis to estimate the potential profitability of MD operations in a dairy farm.

## 2 Methods and Materials

### 2.1 Experiment Setup and Operations

A lab-scale direct contact membrane distillation (DCMD) module (CF042A, Sterlitech, WA) with commercial hydrophobic polyvinylidene fluoride (PVDF) membranes (0.22 μm pore size, 125 μm thickness, GVHP14250, MilliporeSigma, MA) was employed to perform all the experiments. The effective membrane area was 41.1 cm^2^. A recirculation rate of 1.83 mL·s^–1^ was maintained by a pumping system (MFLX7771236, Masterflex, Germany) connecting the chambers of the MD module to the corresponding 1 L reservoir bottles (Figure 1). A water circulator bath (RH-400, Cole-Parmer, IL) was used for maintaining a constant feed solution temperature at 40 °C, while the collector solution was kept at room temperature. The 40 °C as the feed temperature takes advantage of using low-grade heat, and maintaining the collector solution at room temperature reduces energy cost and enhances the feasibility of adopting the MD process in real dairy farms. The water transport is independent of solution pH.^26^

**Figure 1:**
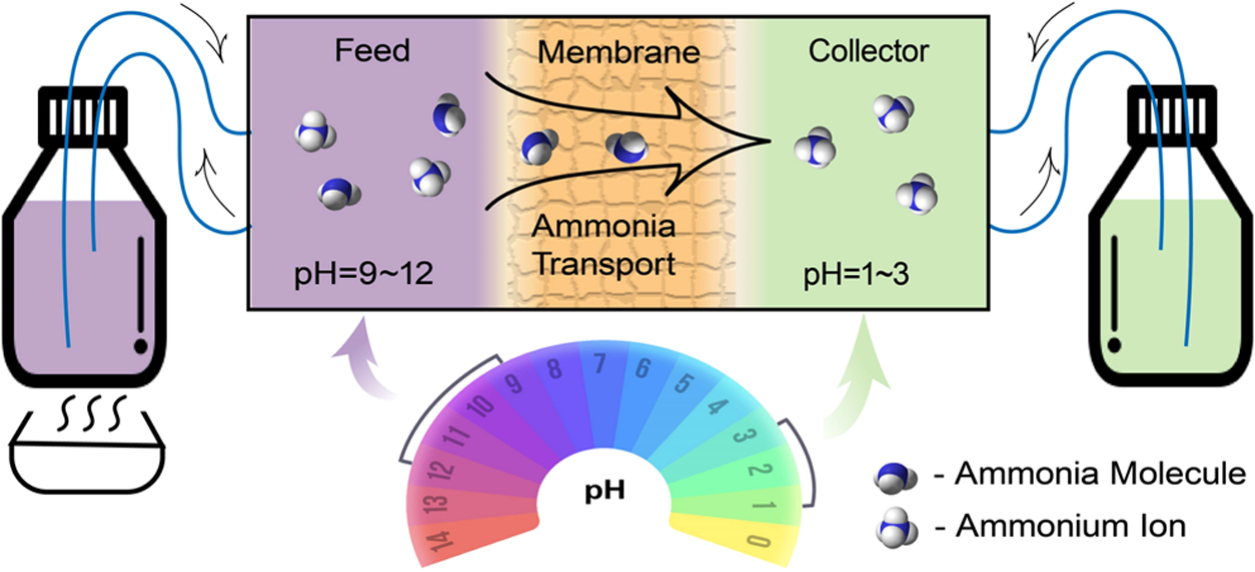
Schematic of direct contact membrane distillation (DCMD) for ammonia recovery from wastewater.

Aqueous NH_4_Cl was used as the feed solution in the DCMD to mimic the ammonia-rich wastewater.^27,28^ A H_2_SO_4_ solution was used as the collector solution and the hydrogen ions enabled the conversion from ammonia molecules to ammonium ions to enhance ammonia transport.^11,15^ To investigate the impact of feed pH and collector pH values on ammonia transport, the initial pH of NH_4_Cl in the feed solution was varied from 9.0 to 12, and the initial pH of the collector solution was set at 1.0, 2.0, and 3.0. The pH adjustment of the chemical solutions was achieved by 1 M NaOH or 1 M H_2_SO_4_ and a pH meter (VSTAR52, Thermo Scientific, MA).

Hydrolyzed urine was also used as the feed solution in DCMD. The urine was collected from the dairy cows at the University of Wisconsin–Madison Dairy Cattle Center and stored for over one month for urea hydrolysis to break down urea into ammonia. The concentration of total ammonia in hydrolyzed urine is listed in Table S1. The pH of the hydrolyzed urine was adjusted to 9.0, 9.5, and 10.0 by NaOH solid pellets prior to use. All chemicals used were purchased from VWR Chemicals, OH. The total ammonia nitrogen (TAN), which consists of ammonia molecules and ammonium ions (TAN = NH_3_ + NH_4_^+^), was measured by the salicylate method using Nitrogen-Ammonia Reagent Set (Hach, CO) with a colorimeter (DR900, Hach, CO).^14,26^

### 2.2 Model Development

A mathematical model was developed to describe the ammonia transport in the MD process. Specifically, we incorporate solution pH in this model to derive the correlation between the solution pH and the ammonia transmembrane behavior. The driving force for ammonia transport in MD is the concentration gradient of ammonia molecules at the two sides of the membrane. The Henry’s Law constant, which is used for partitioning ammonia molecules in solution and in the gas phase, is incorporated into the overall ammonia mass transfer coefficient (eq S1).^11^ Although the temperature dependence of Henry’s Law constant leads to different gas solubilities between the feed and collector sides, the consistent temperature settings used across all MD experiments in this study minimize its impact on the pH effects. Therefore, the observed pH effects are unlikely to be confounded by temperature variations. A mechanistic model will be required to understand how variations in Henry’s Law constants affect the concentration gradient of ammonia across the membrane, which is not the focus of this study. The ammonia mass transfer coefficient in the MD process is calculated based on the ammonia flux and the ammonia concentration gradient across the membrane:^12^

1 $$k_{A}=J_{A}/\left(C_{\mathrm{Af}}-C_{\mathrm{Ac}}\right)$$

where *k*_A_ is the ammonia mass transfer coefficient, *J*_A_ is the ammonia flux, and *C*_Af_ and *C*_Ac_ are the ammonia concentrations at the feed side and the collector side, respectively.

The ammonia concentration at the solution-membrane interface is regulated by both the partitioning between ammonia-ammonium equilibrium in the bulk liquid phase and the gas–liquid partitioning of ammonia at the feed-membrane interface. The NH_3_ concentration in bulk liquid is affected by the pH value:

2 $$\frac{\left[NH_{3}\right]}{\left[NH_{3}\right]+\left[NH_{4}^{+}\right]}=\frac{1}{1+10^{\left(pK_{a}-\mathrm{pH}\right)}}$$

where [NH_3_] and [NH_4_^+^] are the ammonia molecule concentration and ammonium ion concentration, respectively, and p*K*_a_ is the ammonia dissociation constant, which remains unchanged with a fixed solution temperature.

The ammonia recovery efficiency (η) is defined as the ratio of the amount of ammonia in the collector side to the initial amount of ammonia in the feed solution since the volume of the feed solution is identical to that of the collector:

3 $$\eta =\frac{{T\mathrm{an}}_{C,t}}{{T\mathrm{an}}_{F,0}}$$

where TAN_C,*t*_ is the TAN concentration at time *t* in the collector solution, and TAN_F,0_ is the initial TAN concentration in the feed solution.

### 2.3 Preliminary Profit Analysis Calculation

The preliminary profit analysis is based on the generated manure wastewater of the dairy farm and the ammonia recovery rate and capacity of the MD process, which is predicted by the model with the initial feed and collector solution pH values as input parameters (eq S2). The analysis assumes all cow urine from the dairy farm is treated with the 6-h batch MD process to recover the ammonia into the acid collector solution. The recovered ammonia is calculated as the revenue based on the ammonia fertilizer price, and the cost includes membrane cost, acid cost, and base cost (Table S2).

## 3 Results and Discussion

### 3.1 Impact of Solution pH on Ammonia Transport

To understand the effect of solution pH values on ammonia transport, we adjusted the initial pH values on either the feed or collector side. To eliminate the potential interference from competing ions, we did not use a buffer solution to maintain the initial pH value throughout the experiment. Both experimental data and simulation results are presented in Figure 2. In Figure 2A–C, an aqueous solution of 50 mol·m^–3^ NH_4_Cl with varying pH values (i.e., 9, 9.5, 10, 11, and 12) is employed as the initial feed solution and 50 mol·m^–3^ H_2_SO_4_ is the collector solution. In Figure 2D–F, an H_2_SO_4_ solution with varying pH values (i.e., 1, 2, and 3) is applied as the collector solution, while the initial feed solution is 50 mol·m^–3^ NH_4_Cl with a pH value of 11.

**Figure 2:**
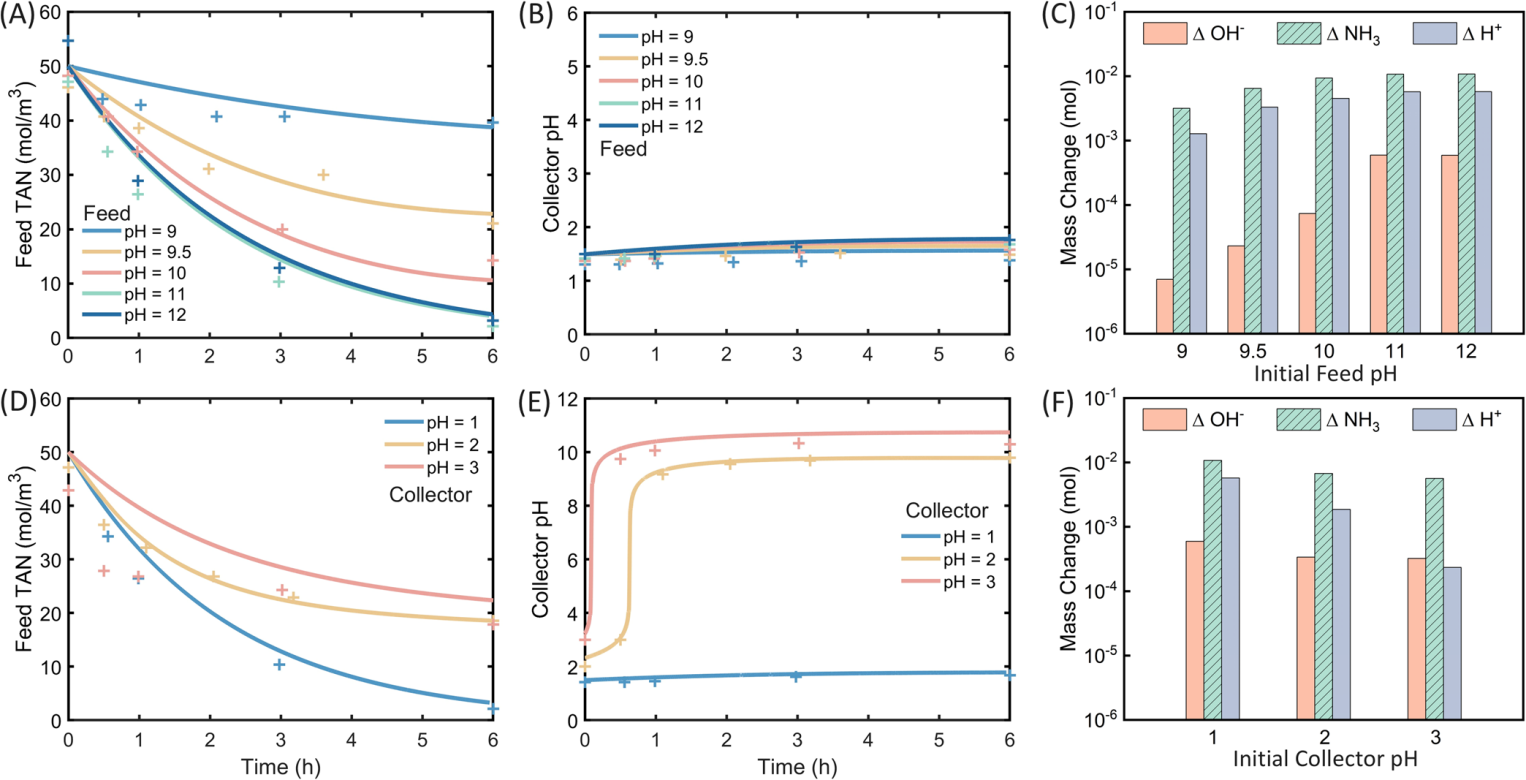
Experiment and simulation results of the membrane distillation (MD) process. For panels A, B, and C, the initial feed pH values of NH_4_Cl solution were set at 9, 9.5, 10, 11, and 12, and the initial collector solution was 50 mol·m^–3^ H_2_SO_4_. For panels D, E, and F, the initial feed pH of NH_4_Cl solution is kept at 11 and the initial collector pH values of H_2_SO_4_ were set at 1, 2, and 3. For panels A, B, D, and E, lines are the simulation results and scatters are the experimental data. (A) Feed total ammonia nitrogen (TAN) concentration and (B) collector pH value as a function of operation time. (C) The calculated mass change of OH^–^ in the feed compartment (orange bar), the amount of transmembrane ammonia (green bar), and H^+^ in the collector compartment (purple) throughout the 6-h operation. (D) Feed TAN concentration and (E) collector pH values as a function of operation time. (F) The calculated mass change of OH- in the feed compartment (orange bar), the amount of transmembrane ammonia (green bar), and H^+^ in the collector compartment (purple) throughout the 6-h operation.

As depicted in Figure 2A, the TAN concentration in the feed solution decreases over time, with solutions having higher initial pH values exhibiting a more rapid decrease and a higher ammonia recovery. With an initial feed solution pH of 9.0, the TAN concentration decreases from 46.1 mol·m^–3^ to 39.6 mol·m^–3^ within 6 h, respectively, with approximately 14% ammonia recovered in the collector. In comparison, the TAN concentration with an initial feed solution pH of 12 decreases from 54.6 mol·m^–3^ to 3.21 mol·m^–3^ within the same duration, indicating a 94% ammonia recovery. The varying recovery efficiency of TAN with different initial feed pH values results from the pH-dependent equilibrium between ammonia molecules and ammonium ions (eq 2). At higher pH values (>10), at least 85% of TAN is in the molecular form (NH_3_), facilitating its volatilization and subsequent transport to the collector side (Figure S1). The simulation results, represented by lines, align well with the experimental data (plus signs), accurately predicting the explicit TAN concentration profile with time. Notably, the TAN concentration profile for initial feed pH values of 11 and 12 slightly overlap, with both simulation results and experimental data showing recovery efficiencies higher than 94%. Thus, further increasing the initial feed solution pH no longer significantly enhances ammonia transport. This observation suggests that excessively high pH levels in the feed solution are unnecessary for efficient ammonia transport since almost all TAN exist as ammonia molecules (NH_3_) at pH 11. However, it is crucial to maintain a high pH value in the feed solution throughout the operation. This is because the transfer of ammonia from the feed to the collector side tends to acidify the feed solution, while simultaneously consuming protons in the collector solution due to the association of ammonia in water. For instance, in the experiment with initial feed pH = 11, the pH of the feed solution decreased to 9.35 at the end of the 6-h experiment, while the pH of the collector solution increased from 1.41 to 1.68 throughout the operation.

On the collector side, we observe a slight pH increase in the collector solution throughout the experiment for all conditions, resulting from the association of ammonia (Figure 2B). Specifically, the final collector pH values range from 1.38 to 1.76, which is about 6.0–18.7% higher than the initial pH values. The mass change of OH^–^ in feed solution, H^+^ in collector solution, and ammonia transmembrane amount are calculated according to eqs S3–5 and they demonstrated a consistent upward trend as the initial feed pH increased (Figure 2C). For instance, when the initial feed pH is 9, the mass change of OH^–^, H^+^, and transmembrane ammonia are 6.98 × 10^–3^, 1.28, and 3.17 mmol, respectively. At an initial feed of 12, these values are 0.59, 5.75, and 10.83 mmol. Additionally, the mass change of OH^–^ and H^+^ is relatively smaller than the ammonia transmembrane amount in each membrane distillation process. For instance, at the initial feed pH of 11, the transmembrane ammonia is 10.77 mmol, whereas the changes of OH^–^ and H^+^ are 0.59 and 5.74 mmol, respectively. These shift in pH levels reflects the dynamic nature of the ammonia transport process and the importance of managing pH throughout the DCMD operation to ensure optimal ammonia recovery efficiency.

A reduction in the pH value on the collector side leads to an increased ammonia transport when the feed pH value is fixed at 11 (Figure 2D–F). With an initial collector pH of 1.0, 2.0, and 3.0, the ammonia recovery efficiencies are 95, 60, and 58%, respectively (Figure 2D). The acidic initial collector solution benefits the ammonia recovery, and a less acidic initial collector solution leads to a lower ammonia recovery efficiency in MD process. Notably, increasing the initial collector pH from 2.0 to 3.0 results in a slight decrease in ammonia recovery efficiency from 60% (TAN from 47.14 to 18.57 mol m^–3^) to 58% (TAN from 42.86 to 17.86 mol m^–3^), indicating further increasing initial collector solution pH cannot effectively suppress ammonia transport. More specifically, in all experimental conditions, the pH value of the collector solution increases throughout the cycle due to the transport of ammonia molecules (Figure 2E). With an initial collector pH of 1.0, the collector pH slightly increases to 1.68 at 6 h. However, with initial collector pH values of 2.0 and 3.0, the final pH values at 6 h are 9.78 and 10.29, respectively. The drastic increase in these two conditions is also captured by the simulation. For MD with an initial collector pH of 2.0, the rapid increase occurs at approximately 0.7 h, and the pH plateau is around 9.6. With an initial collector pH of 3.0, the rapid increase occurs sooner at about 0.1 h and the pH plateau value is approximately 10.5. The rapid increase of pH in the collector could be attributed to the ammonia association in water, which releases hydroxide ions.^29^ When the collector solution is in basic conditions (pH > 9), a significant portion of ammonia exists in the form of ammonia molecules, reducing the concentration difference between the feed and collector side and leading to the formation of a pH plateau. When the initial pH of the collector solution is higher and the acid concentration decreases, it becomes basic more easily due to the decreasing buffer capacity, resulting in a higher pH plateau. Additionally, the mass change of OH^–^, H^+^, and transmembrane ammonia in Figure 2F exhibit trends similar to those in Figure 2C with varying initial feed pH. For an initial collector pH of 1, the mass change of OH^–^, H^+^, and transmembrane ammonia are 0.59, 5.74, and 10.77 mmol, respectively. At an initial collector pH of 3, these values are 0.32, 0.23, and 5.65 mmol. Therefore, the mass changes of OH^–^ and H^+^ are smaller than the ammonia transmembrane amount, confirming the dynamic nature of the ammonia transport process and the importance of solution pH values on ammonia recovery in DCMD.

### 3.2 Ammonia Mass Transfer Coefficient Estimation and Ammonia Recovery Efficiency Prediction

To further elucidate the impact of solution pH values on ammonia transport, the ammonia mass transfer coefficient is calculated in MD according to eq 1. This coefficient reflects the combined effects of molecular diffusion and Knudsen diffusion, which quantify the ammonia molecules collide with each other and with the pore wall, respectively.^12,30^ We derive the ammonia mass transfer coefficient from experimental data with varying feed solution pH initial values (9.0–12.0), collector solution pH initial values (1.0–3.0). Hence, the ammonia mass transfer coefficient quantifies the rate of ammonia transfer per concentration gradient in the experiment, accounting for all contributing factors under the experimental setup. The change of MD reactor design and other operational parameters would lead to a different ammonia mass transfer coefficient value. Based on experimental data, we propose a new empirical equation (eq 4) to describe the effect of pH on the mass transfer coefficient. The correlation between ammonia mass transfer coefficient and pH values in feed and collector solution is hypothetically analogous to the pH regulation on ammonia-ammonium equilibrium (Figure 3A,B). This empirical approach for the ammonia mass transfer coefficient is estimated as follows:

4 $$k_{\mathrm{ammonia}}=k_{A}^{⊖}\cdot \frac{1}{1+10^{\left(C_{1}-{\mathrm{pH}}_{F}\right)}}\cdot \frac{1}{1+10^{\left({\mathrm{pH}}_{C}-C_{2}\right)}}$$

where *k*_ammonia_ is the simulated ammonia mass transfer coefficient, *k*_A_^⊖^ is the reference mass transfer coefficient value, which is determined by experiments. C_1_ and C_2_, which are 9.22 and 2.94, respectively, are the constants to characterize the pH effects on both sides, and pH_F_ and pH_C_ are the pH values in the feed solution and collector solution, respectively. We note that the interferences from other acid/base systems in the solution are neglected.

**Figure 3:**
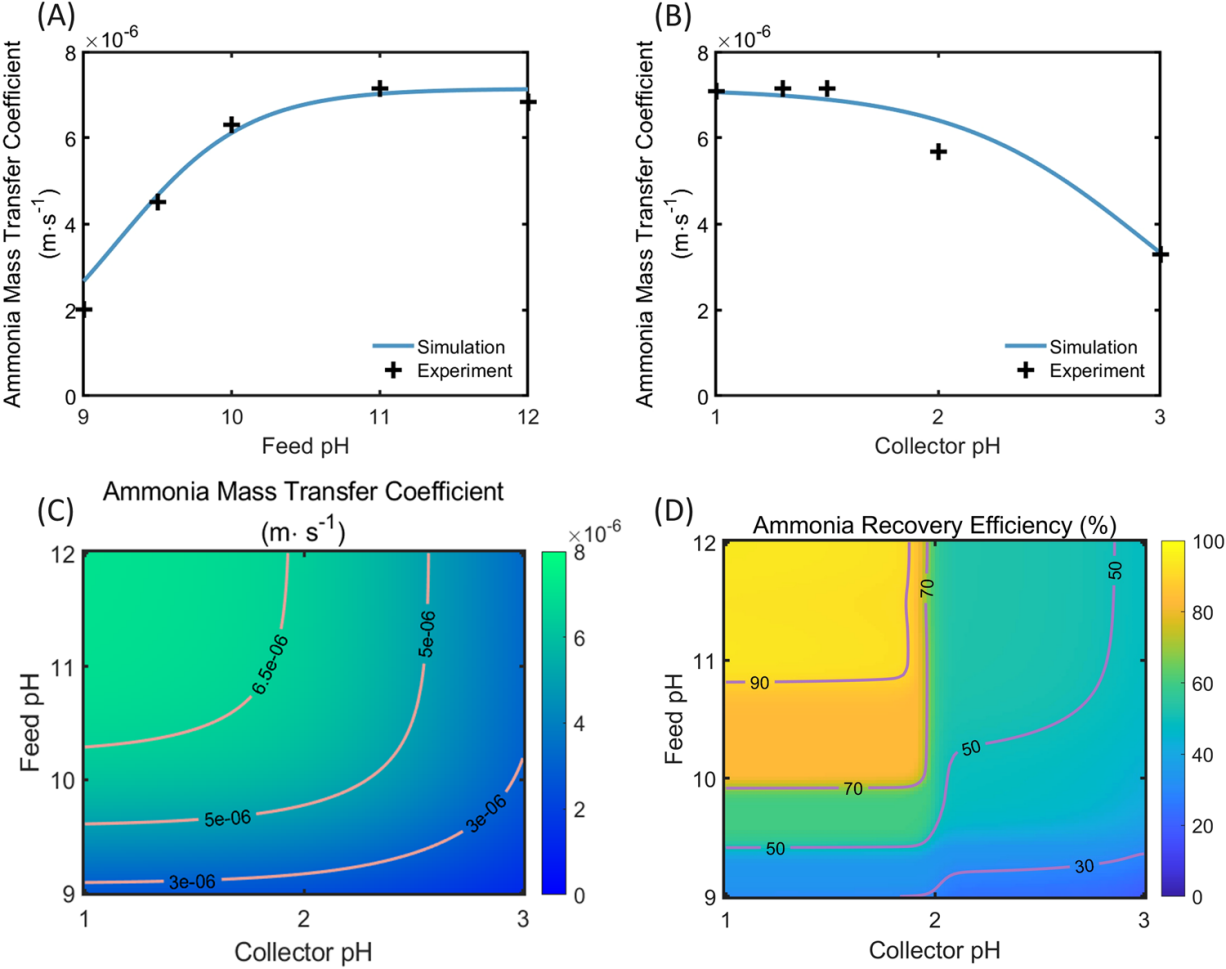
Ammonia mass transfer coefficient experimental data (plus signs) and simulation results (lines) as a function of (A) feed solution initial pH values and (B) collector solution initial values. (C) Ammonia mass transfer coefficient estimation with varying initial feed and collector pH values. The pink contour lines represent certain ammonia mass transfer coefficients. (D) Ammonia recovery efficiency at the membrane distillation operation time of 6 h as a function of initial feed pH and initial collector pH. The purple contour lines show ammonia recovery efficiency of 30, 50, 70, and 90%.

The ammonia mass transfer coefficient is hypothesized to exhibit a similar pH dependency, suggesting that the diffusion of ammonia molecules within the membrane layer is also influenced by the pH values on both sides of the membrane. The practical equation eq 4 fits well in predicting the ammonia mass transfer coefficient with varying initial feed solution pH values. For instance, in Figure 3A, the feed solution initial pH varies from 9 to 12 while the collector solution initial pH remains at a fixed value. The experimental data show that the ammonia mass transfer coefficient increases from 4.50 × 10^–6^ m·s^–1^ to 7.07 × 10^–6^ m·s^–1^ as the initial feed solution pH changes from 9.5 to 11. Correspondingly, predicted ammonia mass transfer coefficient increases from 4.64 × 10^–6^ m·s^–1^ to 7.07 × 10^–6^ m·s^–1^ within the same range. Additionally, when the initial collector solution pH value changes from 1.0 to 3.0 and the initial feed solution pH is kept at 11, the predicted ammonia mass transfer remains consistent with the experimental data. In this series of experiments, the ammonia mass transfer coefficient decreases from 7.07 × 10^–6^ m·s^–1^ to 3.29 × 10^–6^ m·s^–1^ as the initial collector solution pH changes from 1 to 3, while the predicted values drop from 7.07 × 10^–6^ m·s^–1^ to 3.36 × 10^–6^ m·s^–1^. The root-mean-square error (RMSE) between the experimental results and the prediction data is 4.89% based on eq S6. This trend in ammonia mass transfer coefficients indicates that the pH on both sides of the membrane affects the ammonia transmembrane process directly, rather than merely influencing the partition of NH_4_^+^/NH_3_ in the solutions.

We further applied eq 4 to derive the ammonia mass transfer coefficient with initial feed solution pH ranging from 9.0 to 12 and simultaneously varying the initial collector solution pH from 1.0 to 3.0. The pH ranges used in the simulation match the pH range applied in the experiment to make sure that the simulation results are practical and feasible. Figure 3C illustrates the continuous distribution of the ammonia mass transfer coefficient for each specific initial pH value of the feed and collector solutions. The maximum ammonia mass transfer coefficient, 7.07 × 10^–6^ m·s^–1^, occurs with the highest initial feed solution pH and lowest initial collector solution pH, while the ammonia mass transfer coefficient is 1.21 × 10^–6^ m·s^–1^ with initial feed and collector pH of 9 and 3, respectively. The contour lines of ammonia mass transfer coefficients of 3 × 10^–6^ m·s^–1^, 5 × 10^–6^ m·s^–1^ and 6.5 × 10^–6^ m·s^–1^ indicate that higher ammonia mass transfer coefficients are favored under more extreme pH conditions in the feed and collector solutions.

Figure 3D shows how the ammonia recovery efficiency (η) at the end of the 6-h MD process varies with initial feed and collector solution pH values. The simulation of the ammonia recovery efficiency is calculated using 10,000 different initial pH combinations, with 100 discrete values for the initial feed pH, ranging from 9 to 12 in increments of 0.03, and 100 discrete values for the initial collector pH, ranging from 1 to 3 in increments of 0.02. A high initial pH value in the feed solution coupled with a low initial pH in the collector solution leads to an enhanced ammonia recovery efficiency, which shows a similar trend to that of the ammonia mass transfer coefficient. For example, with an initial feed solution pH of 11 and collector solution pH of 1, the recovery efficiency is 86.0%. In contrast, with a lower initial feed solution pH of 10 and a higher collector solution pH of 2, the recovery efficiency is 53.3%. Additionally, the colormap reveals that there is only a marginal increase in ammonia recovery efficiency when the initial feed pH continues to increase beyond approximately 10.8. For instance, the recovery efficiency at 6 h slightly increases from 83.1 to 86.5% as the initial feed solution pH increases from 10.8 to 12, with the initial collector solution pH fixed at 1. The ammonia recovery efficiency distribution with MD operation duration is shown in Figure S2.

The distribution of ammonia recovery efficiency is not as smooth as that of the ammonia mass transfer coefficient, potentially due to the varying NH_4_^+^/NH_3_ partitioning in the solutions. The contour line of 90% recovery efficiency reveals that maintaining a high recovery efficiency necessitates keeping the collector solution pH below approximately 1.9 and the feed solution pH above 10.9, forming an almost rectangular shape in the recovery efficiency distribution. Similarly, the 70% recovery efficiency contour line possesses a rectangular shape, differing from the smooth contour curve of the ammonia mass transfer coefficient distribution. These rectangular shapes of 70 and 90% recovery efficiency imply that in the high recovery efficiency region (>70%), the NH_4_^+^/NH_3_ partition in solutions plays a more critical role than the ammonia mass transfer coefficient. Meanwhile, the 30 and 50% recovery efficiency contour lines resemble a combination of smooth curves and rectangular shapes, representing that both the NH_4_^+^/NH_3_ partition in solutions and the ammonia mass transfer coefficient influence the ammonia recovery efficiency. Therefore, the solution pH governs ammonia recovery efficiency in MD by regulating the NH_4_^+^/NH_3_ partition in solutions and influencing the ammonia transmembrane process by affecting the ammonia mass transfer coefficient, with these factors varying in significance under different operational parameters. A deeper understanding of such mechanisms would assist in enhancing MD performance for ammonia recovery by tuning the operational parameters.

While adjusting both solution pH and solution temperature can impact ammonia transport in MD, we note that pH adjustment is a more practical approach for optimizing MD performance. Increasing the initial feed solution pH from 9.0 to 10 with collector pH fixed at 1 enhances the recovery efficiency from 30.1 to 74.8%, which corresponds to an average ammonia flux increase of 177%. The increase results from the enhanced ammonia mass transfer coefficient from 2.64 × 10^–6^ m·s^–1^ to 6.14 × 10^–6^ m·s^–1^. In contrast, increasing the feed solution temperature from 30 to 65 °C only enhances ammonia flux by 30% and the ammonia mass transfer coefficient by 62%. We note within the same temperature change, water transport in MD would be simultaneously enhanced by 970%, indicating a significant portion of the energy from heated solution is used for water transport rather than ammonia transport.^26^ While MD process can utilize low-grade heat, elevating temperature in the MD process still demands massive energy, making pH adjustment a more effective approach to optimize MD performance.

### 3.3 Ammonia Recovery from Dairy Cow Urine

Liquid cow urine after hydrolysis is used to verify the feasibility of MD for ammonia recovery (Figures 4 and S3). The pH of cow urine is 9.0 after hydrolysis, and the TAN concentration of urine is 480 ± 30 mol·m^–3^, which is higher than that in the initial synthetic manure (∼50 mol·m^–3^). Three pH values (i.e.,9.0, 9.5, and 10.0) in the feed solution are investigated, while the pH of the initial collector solution is set at 1.0. The total ammonia nitrogen (TAN) concentrations in the feed solution steadily decrease with time across all initial feed solution pH values (Figure 4A). The trend of the feed TAN concentration in the real urine experiments aligns with that in the synthetic manure experiment (Figure 2A). With the initial feed pH values of 9.0, 9.5, and 10.0, the feed TAN concentrations decrease to 375, 264, and 161 mol·m^–3^, respectively. We still observe a good fitting of simulation TAN concentrations with experimental data, with an RMSE of 3.14%, verifying that the mathematical model is valid for real urine with high TAN concentrations.

**Figure 4:**
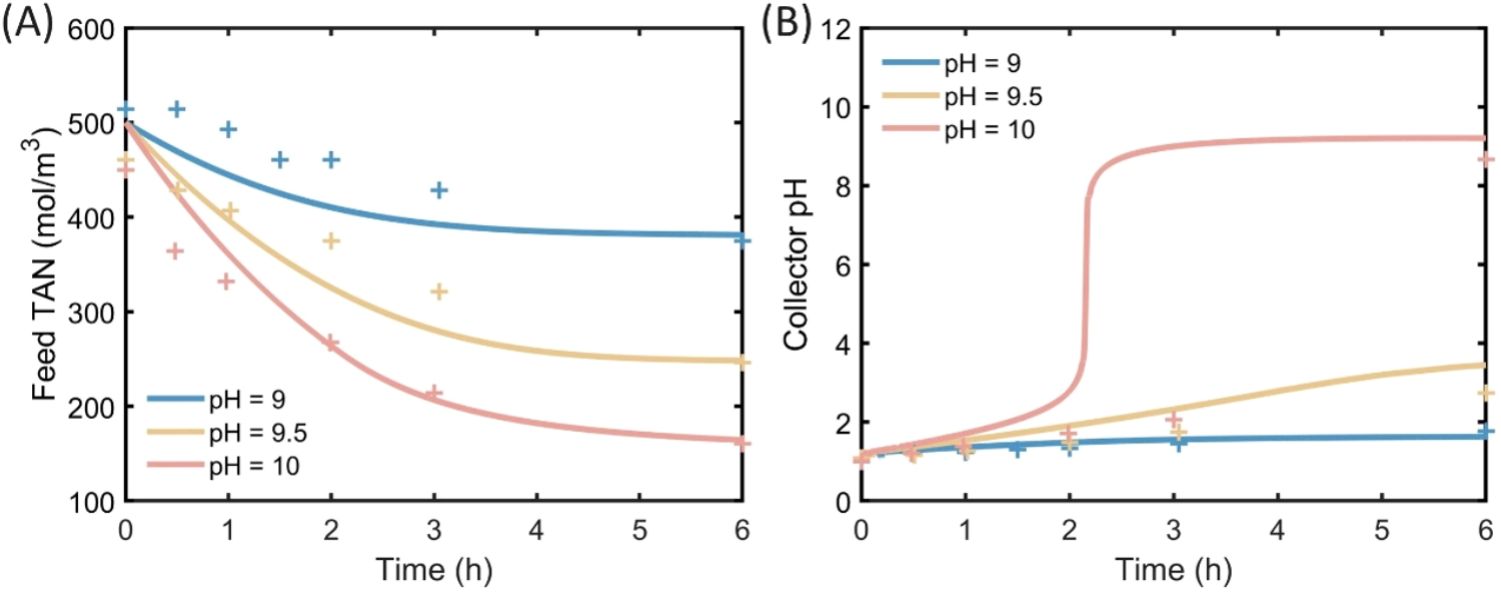
Ammonia recovery experimental results (plus signs) and simulation data (lines) by batch membrane distillation from dairy cow urine. Dairy cow urine after pH adjustment is used as the feed solution and the initial collector solution pH is adjusted to 1. (A) Feed total ammonia nitrogen concentration change with experiment time. (B) Collector pH changes with experiment time.

The collector pH change trend in the urine MD experiment (Figure 4B) corresponds with that observed in synthetic manure (Figure 2B). With the initial feed pH values of 9.0, 9.5, and 10.0, the final collector pH values increase to 1.77, 2.74, and 8.66, respectively. Although the pH simulation data generally aligns with the experiment result, there remains some discrepancies between experimental values and simulation predictions. Real urine is a complex mixture of aqueous ammonia with other organic and inorganic chemicals, which exhibit a great pH buffer capacity. In contrast, synthetic manure is essentially an aqueous solution of NH_4_Cl and NaOH, with a predictable pH value as determined by the mathematical model. Therefore, the different buffer capacities between synthetic manure and real urine potentially cause deviations in the collector pH value. Overall, the model performs well in simulating ammonia transport across the membrane in both synthetic manure wastewater and real urine. Further calibration of the pH buffering capacity in real urine could improve the accuracy of the proposed mathematical model.

### 3.4 Preliminary Profit Analysis

Recovering ammonia from manure wastewater facilitates minimizing ammonia emissions from lagoons, preventing soil and groundwater contamination, and reducing cost for fertilizer costs.^3^ Based on our calculation, approximately 90% of the ammonia from urine could be recovered by a 6-h batch MD process when the initial pH of feed solution is adjusted to 10.9 and the initial pH of collector solution is set at 1.9. The techno-economic analysis could quantify the benefits of ammonia recovery and provide preliminary revenue and cost data for future scaling-up and commercialization of this technology. A preliminary profit analysis is conducted at a dairy farm with 2000 cows where a 6-h batch MD process is used to recover ammonia as fertilizer (Figure 5A). The annual gross profit is estimated based on varied initial solution pH values, and the calculation is based on the recovered ammonia as revenue while the input additional acid/base and membrane are considered as costs. We maintain consistency between experiments and profit analysis to ensure a practical and feasible profit estimation, as considering other parameters, including varying operation duration and changing to continuous mode, would require additional model calibration. The cost of maintaining feed solution temperature at 40 °C is excluded due to the dominant role of pH values and the potential availability of low-grade heat in practical settings.

**Figure 5:**
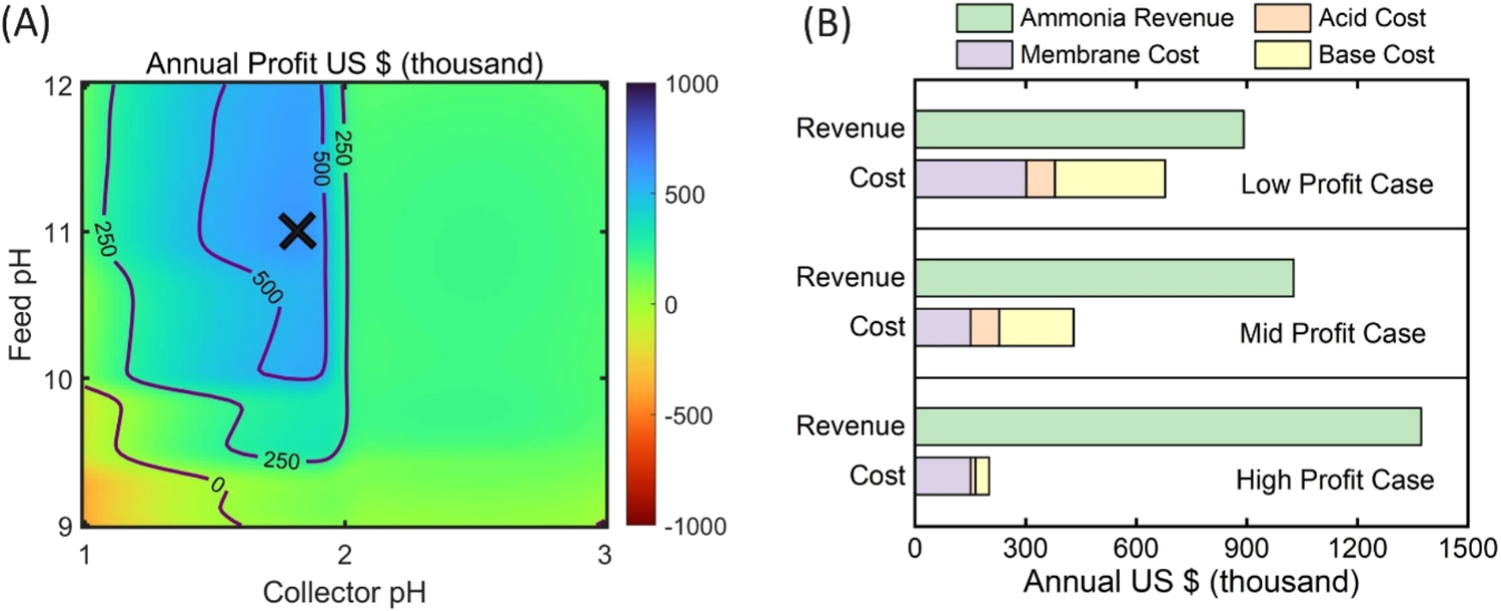
Ammonia recovery by membrane distillation profit analysis based on a dairy farm with 2000 cows. (A) Annual profit distribution with varying initial feed and collector pH. The purple contour lines show certain annual profit values. The black cross represents the maximum estimated annual profit of ∼598,000$ with initial feed and collector pH of 11.01 and 1.82, respectively. (B) Annual revenue and cost from the profit analysis based on the operation configuration of the maximum annual profit (black cross in panel A). Revenues and costs are calculated considering low, mid, or high chemical and membrane prices, deriving low, mid, and high profit case scenarios for ammonia recovery, respectively.

The revenue of ammonia as fertilizer and the cost of additional acid/base input as pH adjustments change accordingly. Although the most extreme initial solution pH values lead to the highest ammonia recovery efficiency, the massive acid/base input demands huge chemical costs, diminishing the gross profit. The maximum possible annual profit of this batch MD process is ∼$598,000 with the initial feed and collector solution pH of 11.01 and 1.82, respectively (denoted as a cross in Figure 5A). The annual profit of $0 contour line implies the minimum requirement of initial pH configuration for possible profit.

To further investigate each component of the gross profit, a revenue and cost analysis is conducted, taking the impact of chemical and membrane prices into account (Figure 5B). The initial pH configuration is the maximum annual profit point, with the initial feed and collector solution pH of 11.01 and 1.82, respectively (the cross in Figure 5A). The optimal initial pH configuration with the maximum gross profit estimation changes due to the ammonia recovery efficiency and the revenue and cost fluctuation. Seeking and updating the optimal configuration parameters would be critically beneficial in real MD applications since the MD operation needs to respond to the chemical market price, which oscillates temporally and spatially, causing significant differences between cases. The annual profit of low, mid, and high cases is $213,000, $598,000, and $1,174,000, respectively (Figure S4). The cost breakdown demonstrates that membrane is a major expense in all scenarios, suggesting that reducing membrane cost could significantly enhance the feasibility of applying MD for ammonia recovery in dairy farms. Although the actual operation process requires consideration of additional factors beyond this preliminary profit model — such as equipment investment, operation and maintenance cost, and heat and electricity consumption — this analysis highlights the comparison between ammonia revenue and chemical costs as a valuable insight for the potential real-world application of MD in dairy farms.

## 4 Environmental Implications

Improving ammonia transport in the direct contact membrane distillation (DCMD) process is essential for ammonia recovery from wastewater. In this study, we comprehensively investigated the effect of solution pH values on ammonia transport and recovery efficiency in DCMD, utilizing both experimental and modeling approaches. Unlike previous research that primarily focused on the pH effect on a single side of the membrane distillation (MD) process, our study assessed MD performance by examining pH levels on both the feed and collector sides, integrating findings from experimental data and simulation outcomes. Our results revealed that pH plays a dual role in ammonia recovery: it not only controls the ammonia-ammonium equilibrium in the solutions but also significantly influences the ammonia mass transfer coefficient. When compared to previous studies that focused on the impact of temperature, we found that pH adjustment plays a more substantial role in affecting both the mass transfer coefficient and the recovery efficiency. Moreover, our simulations of ammonia recovery efficiency facilitate the optimization of MD performance, allowing us to fine-tune the pH range in the solutions and the duration of operation.

While this study unraveled the critical role of solution pH on ammonia transport in the MD process, several critical issues remain unresolved and warrant further investigation. One such issue is the behavior of ammonia transport under extreme pH conditions, which is currently not well understood. In addition, the effect of concentration polarization is incorporated into the empirical ammonia mass transfer coefficient. Decoupling the concentration polarization from ammonia transport would require a mechanistic model. Furthermore, there are concerns regarding the potential adverse effects of these conditions on membrane stability, which could impact the process’s long-term viability. Before industrial application, the model needs further refinement by considering the operational cost, pretreatment, and post-treatment to determine an optimal duration time. Incorporating the cost of pH adjustment and conducting a thorough technical-economic analysis would provide a more holistic view of the process. Additionally, recovering ammonia from urine and livestock wastewater prevents ammonia contamination to soil, groundwater, and atmosphere, while this economic analysis excludes such environmental impact of reducing ammonia emissions. Typically, 60% of total ammonia emissions to the atmosphere are from dairy farms.^9^ Quantifying the monetary benefit of preventing ammonia emissions could further improve the estimated profit, and preventing each kilogram of ammonia emission to the atmosphere is equivalent to saving $100 according to annual health costs.^31^

## Supplementary Material



## Notes

Dairy cow urine collection and characterization (Table S1); chemical and membrane prices for the midprofit case calculation (Table S2); ammonia-ammonium partition as a function of solution pH (Figure S1); ammonia recovery efficiency simulation (Figure S2); ammonia recovery performance from dairy cow urine (Figure S3); profit analysis with different profit scenarios (Figure S4); ammonia mass transfer coefficient calculation with Henry’s Law (eq S1); calculation of preliminary profit analysis (eq S2); calculation of performance matrices characterization (eqs S3–S5); calculation of root mean square error (eq S6) (PDF)

## References

[1] Mrówczyńska-Kamińska A., Bajan B., Pawlowski K. P., Genstwa N., Zmyslona J. (2021). Greenhouse
gas emissions intensity of food production systems and its determinants. PLoS One.

[2] Gao Y., Cabrera Serrenho A. (2023). Greenhouse
gas emissions from nitrogen fertilizers
could be reduced by up to one-fifth of current levels by 2050 with
combined interventions. Nat. Food.

[3] Lemaire G., Franzluebbers A., Carvalho P. C. d. F., Dedieu B. (2014). Integrated crop–livestock
systems: Strategies to achieve synergy between agricultural production
and environmental quality. Agric., Ecosyst.
Environ..

[4] Gu B., Zhang L., Van Dingenen R., Vieno M., Van Grinsven H. J., Zhang X., Zhang S., Chen Y., Wang S., Ren C., Rao S., Holland M., Winiwarter W., Chen D., Xu J., Sutton M. A. (2021). Abating ammonia
is more cost-effective than nitrogen oxides for mitigating PM2.5 air
pollution. Science.

[5] Erisman J. W. (2021). How ammonia
feeds and pollutes the world. Science.

[6] Ornes S. (2021). Core Concept:
Green ammonia could produce climate-friendly ways to store energy
and fertilize farms. Proc. Natl. Acad. Sci.
U.S.A..

[7] Bodirsky B. L., Popp A., Lotze-Campen H., Dietrich J. P., Rolinski S., Weindl I., Schmitz C., Muller C., Bonsch M., Humpenoder F., Biewald A., Stevanovic M. (2014). Reactive nitrogen
requirements to feed the world in 2050 and potential to mitigate nitrogen
pollution. Nat. Commun..

[8] Cruz H., Luckman P., Seviour T., Verstraete W., Laycock B., Pikaar I. (2018). Rapid removal of ammonium
from domestic
wastewater using polymer hydrogels. Sci. Rep..

[9] Aguirre-Villegas H. A., Besson C., Larson R. A. (2024). Modeling
ammonia emissions from manure
in conventional, organic, and grazing dairy systems and practices
to mitigate emissions. J. Dairy Sci..

[10] Laganà F., Barbieri G., Drioli E. (2000). Direct contact membrane distillation:
modelling and concentration experiments. J.
Membr. Sci..

[11] McCartney S. N., Williams N. A., Boo C., Chen X., Yip N. Y. (2020). Novel Isothermal
Membrane Distillation with Acidic Collector for Selective and Energy-Efficient
Recovery of Ammonia from Urine. ACS Sustainable
Chem. Eng..

[12] Jiang H., Straub A. P., Karanikola V. (2022). Ammonia Recovery
with Sweeping Gas
Membrane Distillation: Energy and Removal Efficiency Analysis. ACS ES&T Eng..

[13] El-Bourawi M. S., Khayet M., Ma R., Ding Z., Li Z., Zhang X. (2007). Application of vacuum
membrane distillation for ammonia removal. J.
Membr. Sci..

[14] Yang K., Du H., Qin M. (2023). Solar enhanced membrane
distillation for ammonia recovery. J. Membr.
Sci. Lett..

[15] Zarebska A., Nieto D. R., Christensen K. V., Norddahl B. (2014). Ammonia recovery from
agricultural wastes by membrane distillation: fouling characterization
and mechanism. Water Res..

[16] Qu D., Sun D., Wang H., Yun Y. (2013). Experimental study of ammonia removal
from water by modified direct contact membrane distillation. Desalination.

[17] Duong T., Xie Z., Ng D., Hoang M. (2013). Ammonia removal
from aqueous solution
by membrane distillation. Water Environ. J..

[18] Xu K., Qu D., Zheng M., Guo X., Wang C. (2019). Water Reduction and
Nutrient Reconcentration of Hydrolyzed Urine via Direct-Contact Membrane
Distillation: Ammonia Loss and Its Control. J. Environ. Eng..

[19] Zhu Y., Chang H., Yan Z., Liu C., Liang Y., Qu F., Liang H., Vidic R. D. (2024). Review
of ammonia recovery and removal
from wastewater using hydrophobic membrane distillation and membrane
contactor. Sep. Purif. Technol..

[20] Bates R. G., Pinching G. D. (1949). Acidic dissociation constant of ammonium ion at 0 to
50 C, and the base strength of ammonia. J. Res.
Natl. Bur. Stand..

[21] Vo T.-D.-H., Bui X.-T., Dang B.-T., Nguyen T.-T., Nguyen V.-T., Tran D. P. H., Nguyen P.-T., Boller M., Lin K.-Y. A., Varjani S., Show P. L. (2021). Influence of organic loading rates
on treatment performance of membrane bioreactor treating tannery wastewater. Environ. Technol. Innovation.

[22] Hu Y., Loh C. Y., Xie M., Chen G., Huang M., Qiao J. (2024). Ammonia recovery via
direct contact membrane distillation: Modeling
and performance optimization. J. Environ. Manage..

[23] Tarpeh W. A., Barazesh J. M., Cath T. Y., Nelson K. L. (2018). Electrochemical
Stripping to Recover Nitrogen from Source-Separated Urine. Environ. Sci. Technol..

[24] Wang R., Yang K., Wong C., Aguirre-Villegas H., Larson R., Brushett F., Qin M., Jin S. (2024). Electrochemical
ammonia recovery and co-production of chemicals from manure wastewater. Nat. Sustainability.

[25] Simoni G., Kirkebæk B. S., Quist-Jensen C. A., Christensen M. L., Ali A. (2021). A comparison of vacuum
and direct contact membrane distillation for
phosphorus and ammonia recovery from wastewater. J. Water Process Eng..

[26] Yang K., Qin M. (2024). Understanding Ammonia
and Water Transport in Direct Contact Membrane
Distillation toward Selective Ammonia Recovery. ACS ES&T Eng..

[27] Fang K., He W., Peng F., Wang K. (2020). Ammonia recovery from concentrated
solution by designing novel stacked FCDI cell. Sep. Purif. Technol..

[28] Obaja D., Mace S., Mata-Alvarez J. (2005). Biological
nutrient removal by a
sequencing batch reactor (SBR) using an internal organic carbon source
in digested piggery wastewater. Bioresour. Technol..

[29] Hales J. M., Drewes D. R. (1979). Solubility of ammonia in water at low concentrations. Atmos. Environ..

[30] Cai J., Guo F. (2017). Study of mass transfer
coefficient in membrane desalination. Desalination.

[31] Paulot F., Jacob D. J. (2014). Hidden cost of U.S.
agricultural exports: particulate
matter from ammonia emissions. Environ. Sci.
Technol..

