# Supplementary material for: Dual Effect of Solution pH on Ammonia Recovery in Membrane Distillation – Influence on pH Partitioning and Mass Transfer Coefficient
Source: ACS ES T Eng. 2025 Jul 28;5(11):2855–63. doi: 10.1021/acsestengg.5c00332 (PMC12624731; doi:10.1021/acsestengg.5c00332)
Supplement: Supplementary file 1 [file ee5c00332_si_001.pdf]

Supporting Information for

Dual Effect of Solution pH on Ammonia Recovery in Membrane Distillation – Influence on pH Partitioning and Mass Transfer Coefficient

*Kai Yang, Benjamin Michael Simplot, and Mohan Qin**

Department of Civil and Environmental Engineering, University of Wisconsin–Madison, Madison, Wisconsin 53706, USA

* Corresponding author. E-mail: mohan.qin@wisc.edu

1. Dairy cow urine collection and characterization

The urine was collected from the Dairy Cattle Center on campus, and dairy cows were stimulated to excrete urine. The collected urine was stored in a fume hood at room temperature and the urea hydrolysis process generated aqueous ammonia and high pH (~9.0). The detailed ammonia concentration change during the hydrolysis process are list in Table S1.

Table S1: Concentration of total ammonia nitrogen in hydrolyzed dairy cow urine.

Days	Total ammonia nitrogen concentration (mg-N/L)
6	450
7	600
13	1000
14	2600
21	6400
26	7200
35	6750

2. Ammonia mass transfer coefficient calculation with Henry's Law

The ammonia gas molecules transport across the MD membrane and the ammonia gas molecule concentration (or partial pressure, Paf,gas) gradient in vapor phase is the driving force of ammonia transmembrane movement. Since the ammonia molecules in gas phase and liquid phase follow the Henry's Law and the Henry's Law constant (H_A) is identical for feed side and collector side, the Equation 1 could be obtained from the following:

$$J_A = k \cdot \left(P_{Af,gas} - P_{Ac,gas}\right) = \frac{k}{H_A} \cdot \left(C_{Af} - C_{Ac}\right) = k_A \cdot \left(C_{Af} - C_{Ac}\right) \quad \text{(S1)}$$

where J_A is the ammonia flux, k is the mass transfer coefficient for ammonia at gas phase, k_A is the overall ammonia mass transfer coefficient, which incorporates the Henry's Law constant. Therefore, we used the k_A as the overall ammonia mass transfer coefficient and Eq. 1 and the driving force in Eq. 1 is ammonia concentration in solution.

3. Calculation of preliminary profit analysis

The estimated profit of operating MD in a 2000 cows dairy farm towards ammonia recovery is calculated by:

$$\text{Annual Profit} = T_{amm} \cdot \eta_{amm} \cdot P_{amm} - N \cdot (C_{membrane} - C_{acid} - C_{base}) \quad \text{(S2)}$$

where T_{amm} is the total ammount of the ammonia in the 2000 cows urine, η_{amm} is the predicted ammonia recovery efficiency based on the initial pH values of the feed and collector solution, P_{amm} is the market price of ammonia fertilizer, N is the number of the MD reactors needed to be capable of treating urine from 2000 cows in the dairy farm, $C_{membrane}$ is the cost of the membrane, C_{acid} and C_{base} is the cost of acid and base chemicals for adjusting the pH of the initial feed and collector soltuion in each reactor. Such profit estimation is an optimistic outlook for the MD process.[1] Therefore only consider the chemical and membrane cost, focusing on comparsion between ammonia revenue and chemical cost and potentially provide insight into real worlk application of MD in dairy farms.

The chemical and membrane prices used for profit analysis are obtained in July 2024 are listed as Table S2.

Table S2: Chemical and membrane prices for the mid-profit case calculation

Product	Source/Company	Unit price	
PVDF	Tisch scientific	43.5	$/m^2
NaOH	Alliance Chemical	0.153	$/mol
H_2SO_4	ChemWorld	0.185	$/mol
Ammonia	USDA Product Cost Report 6/14/2024	0.068	$/mol

4. Calculation of performance matrices characterization

The mass change of OH^- ($\Delta\ OH^-$) in the feed compartment is calculated by:

$$\Delta\, OH^- = V_F \cdot (10^{pH_{initial}-14} - 10^{pH_{final}-14}) \tag{S3}$$

where V_F is the volume of the feed compartment, $pH_{initial}$ and pH_{final} are the pH value at the initial and final stage of the feed solution, respectively.

The amount of transmembrane ammonia ($\Delta\, NH_3$) is calculated by:

$$\Delta\, NH_3 = V_F \cdot (C_{initial} - C_{final}) \tag{S4}$$

where $C_{initial}$ and C_{final} are the total ammonia nitrogen concentration at the initial and final stage of the feed solution, respectively.

The mass change of H^+ ($\Delta\, H^+$) in the collector compartment is calculated by:

$$\Delta\, H^+ = V_C \cdot (10^{-pH_{initial}} - 10^{-pH_{final}}) \tag{S5}$$

where V_C is the volume of the collector compartment, $pH_{initial}$ and pH_{final} are the pH value at the initial and final stage of the collector solution, respectively.

5. Calculation of root mean square error

The root mean squre error (RMSE) between the experiment results and simulation data is calculated by:

$$RMSE = \frac{\sqrt{\sum \frac{1}{n} \cdot \left(y_{i,exp} - y_{i,pre}\right)^2}}{\max\{y_{i,exp}\}} \tag{S6}$$

where $y_{i,exp}$ and $y_{i,pre}$ are the experiment value and simulation value, n is the total number of the $y_{i,exp}$, and max $\{y_{i,exp}\}$ is the max value of all the $y_{i,exp}$.

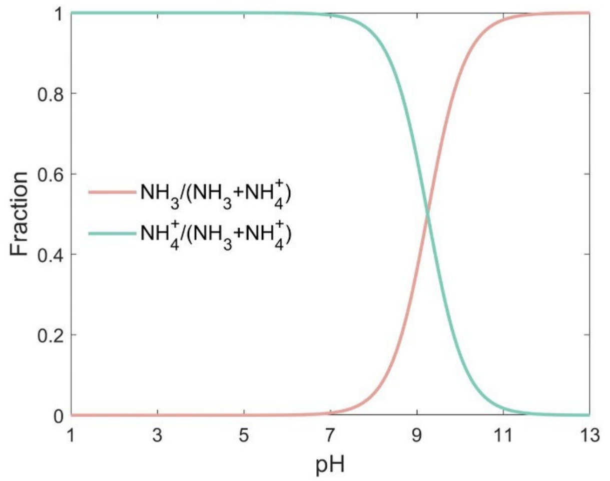

Figure S1. Ammonia-ammonium partition as a function of solution pH.

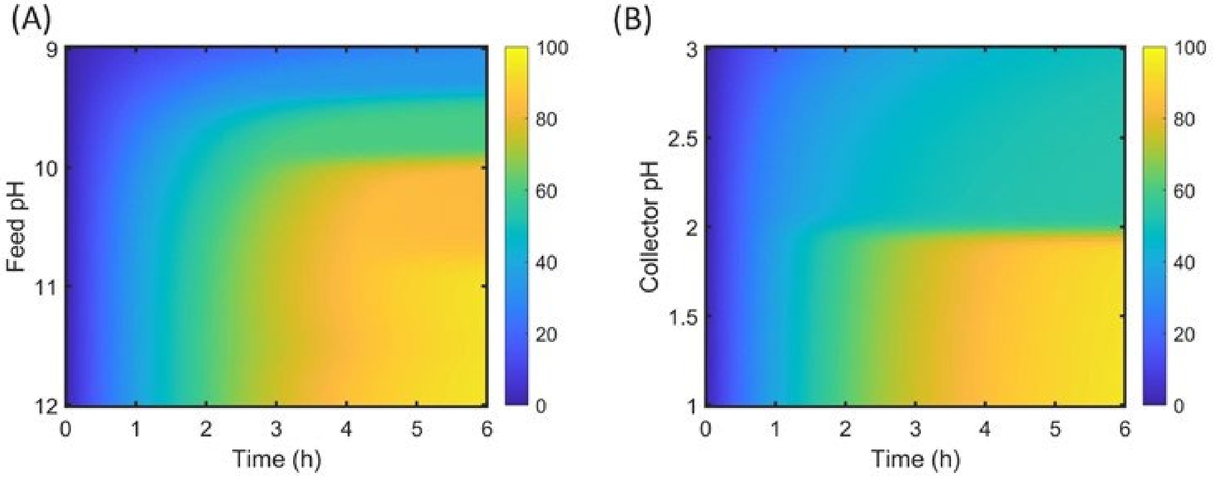

Figure S2. Simulation of ammonia recovery efficiency (%) as a function of time at different initial (A) feed pH and (B) collector pH. For (A), the initial collector pH is 1. For (B), the initial feed pH is 11.

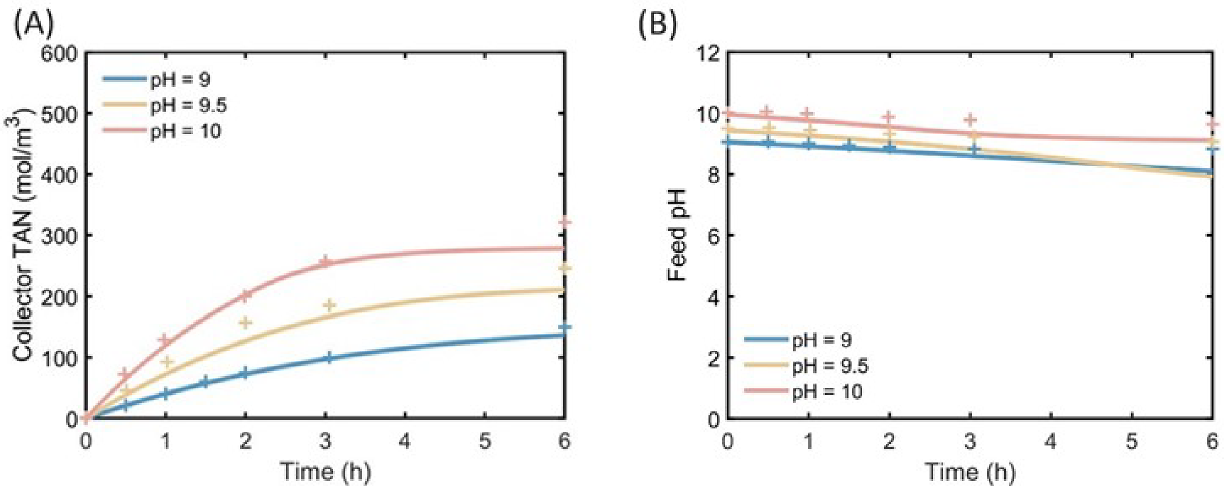

Figure S3. Ammonia recovery performance from dairy cow urine. (A) Collector total ammonia nitrogen (TAN) concentration variation within 6 hours experiment. (B) Feed manure pH variation. The initial feed pH is adjusted to 9, 9.5, and 10. The initial collector pH is 1. Cross represents the experimental data and line represents the simulation results.

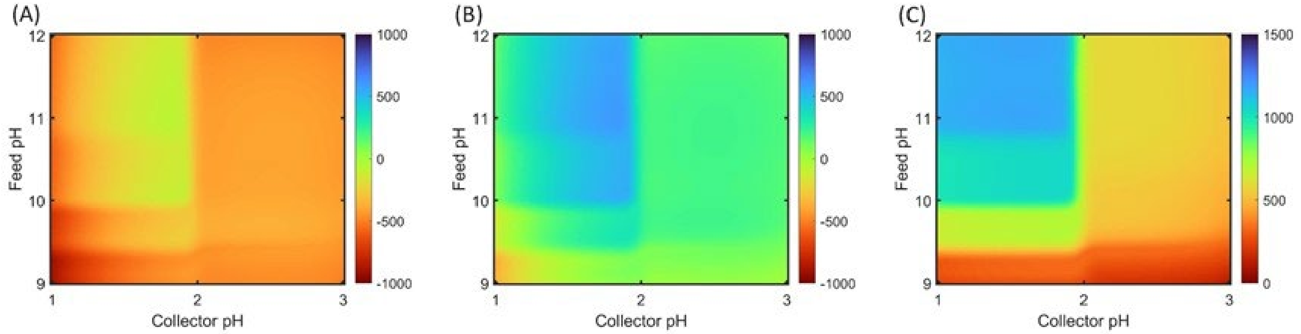

Figure S4. Profit analyses of membrane distillation ammonia recovery based on a dairy farm with 2000 cows. Profit distribution with different pH conditions at: (A) low profit scenario with high chemical costs and low revenues; (B) mid profit scenario with moderate chemical costs and mid revenues; (C) high profit scenario with low chemical costs and high revenues.

Reference

1. Patel, S. K.; Lee, B.; Westerhoff, P.; Elimelech, M., The potential of electrodialysis as a cost-effective alternative to reverse osmosis for brackish water desalination. *Water Res* **2024,** *250*, 121009.
